# Gaze improves working memory for verbal items

**DOI:** 10.12688/openreseurope.21720.1

**Published:** 2025-11-20

**Authors:** Vinoprasath Shivakumar, Ljerka Ostojić, Edward Legg

**Affiliations:** 1 Division of Cognitive Sciences, Faculty of Humanities and Social Sciences, University of Rijeka, Rijeka, Primorje-Gorski Kotar, 51000, Croatia; 2 Department of Psychology, Faculty of Humanities and Social Sciences, University of Rijeka, Rijeka, Primorje-Gorski Kotar, 51000, Croatia; 3 Centre For Mind and Behaviour, University of Rijeka, Rijeka, Primorje-Gorski Kotar County, 51000, Croatia

**Keywords:** gaze cueing, working memory, joint attention, attention orienting

## Abstract

**Background:**

Previous studies have shown that stimuli that another individual looks at are better remembered than stimuli that are not looked at, suggesting that joint attention improves memory. However, these previous studies have differed in the type of memory being tested and the type of content that is to-be-remembered: while effects of joint attention on long-term memory were tested with verbalizable stimuli, effects on working memory have only been tested with visual stimuli such as colour. Thus, the aim of the current study is to extend these previous findings and investigate whether joint attention improves working memory for verbalizable stimuli.

**Methods:**

Participants were first presented with an image of a face with eyes that gazed either to the left or to the right, after which a grid of 4 letters (2x2) was shown. On half of trials, this grid with letters was shown in the same direction that was gazed at, and in the other half of the trials, in the other direction. After a retention interval (1000 ms), participants were shown a letter in the centre of the screen and asked to judge whether they have seen this letter as part of the grid shown before.

**Results:**

Our results revealed that participants’ judgements about whether they had previously seen the letter was more accurate for letters that had been gazed at than letters that had not been gazed at. In contrast, participants’ reaction times were not influenced by whether the letter had been gazed at.

**Conclusions:**

Our findings suggest that joint attention can improve working memory for verbalizable stimuli such as letters.

## Introduction

The physiology of human eyes with their large white sclera and dark iris helps provide a clear signal of the direction in which an individual is looking ( Kobayashi & Kohshima, 1997). A large body of research using manipulations to face and eye stimuli that reflect shifts in the iris position relative to the sclera have demonstrated that humans are remarkably sensitive to the direction of another’s gaze. Already 3-month old infants shift their own attention in the direction signalled by images of eyes looking to the left or right placed on an image of a face ( Hood *et al.*, 1998). For adults, such shifts in attention appear to have some degree of automatization, appearing in situations where gaze fails to predict a target’s location and when participants are placed under cognitive load ( Friesen & Kingstone, 1998; Visser & Roberts, 2018).

This sensitivity to the direction of another’s gaze further allows two individuals to coordinate their attention toward the same external stimulus. The benefits of such ‘joint attention’ have been the subject of extensive discussion ( Kwisthout *et al.*, 2008; Moore *et al.*, 2014; Tomasello, 1995). One strand of research on the downstream consequences of joint attention focuses on processes that have played key roles in the discussion about the evolution of joint attention – such as the impact of joint attention on word learning ( Hirotani *et al.*, 2009) and on conveying information about preferences ( Bayliss *et al.*, 2006; Bayliss *et al.*, 2007). A second strand of research investigates the impact of joint attention on domain-general processes that could be affected by an individual’s previous level of attention, such as working memory and long-term memory. Dodd *et al.* (2012) demonstrated the effect of gaze cues on long term memory. Participants first saw a schematic face that did not have pupils/irises. Then, the pupils/irises were shown, making the face appear to look to the left or to the right. Finally, a word was shown either to the left or to the right side of the face, such that the word appeared either on the side that the eyes were looking toward or on the opposite side. When asked to recall the words at the end of the experiment, participants remembered words better when they had appeared in the location that the central face had been looking at, suggesting that joint attention may improve long term memory of written words. Complementing these results, Gregory and Jackson (2017) found that participants showed higher accuracy of working memory for the gazed at items than the non-gazed at items. Here, participants were presented with a grid of 4, 6 or 8 coloured squares either on the side that a central face gazed toward or on the opposite side. There were thus two critical differences between these two studies. First, in Dodd *et al.* (2012), the to-be-remembered items were words, and in Gregory and Jackson (2017), they were square patches of colour – such that in the former, the memory test was based on verbalizable information, and in the latter, the memory test was based on visual information. Second, in Dodd *et al.* (2012), the test was conducted at the end of the presentation of all to-be-remembered items, and in Gregory and Jackson (2017), the test was conducted 1000 ms after the offset of the to-be-remembered items – such that in the former, long-term memory was assessed, and in the latter, working memory was assessed. Consequently, current evidence indicates that joint attention enhances long-term memory for verbalizable information (i.e., words) and working memory for visual information (i.e., colour).

Before these results can be taken as an indication that joint attention produces improvements to different types of memory across different stimuli, it is crucial to consider that processes involved in storing verbal and non-verbal information in working memory are commonly proposed to be separate ( Baddeley, 2003). While verbal information is likely being stored within the phonological loop, with information decaying rapidly unless subject to a maintenance process such as subvocal rehearsal ( Camos *et al.*, 2009; although see Oberauer, 2019), non-verbal information is thought to be stored in the visuo-spatial sketchpad and is also maintained through maintenance strategies which may involve allocating attention – in the absence of the stimulus - to the spatial location of stimuli that need to be remembered ( Awh *et al.*, 2006). Considering the results of both Dodd *et al.* (2012) and Gregory and Jackson (2017), this raises the question of whether joint attention only improves long-term memory for verbalizable stimuli or whether the improvement for such stimuli is also observed for working memory. Thus, the aim of the current study was test whether joint attention improves verbal working memory performance using a modified procedure of Gregory and Jackson (2017) such that the to-be-remembered items were letters.

## Methods

### Participants

A power analysis using the R package ‘pwrrs’ indicated that a sample size of 34 was needed to detect a medium sized effect (cohen’s d = 0.5) with a power of 0.8 using a paired sample t-test with the significance level set at .05. Thirty-six participants with a mean age of 38 (SD = 11.4) were recruited using prolific.com and were compensated £2.25 for taking part in the study. The study was approved by the Ethics Committee for Research at the Faculty of Humanities and Social Sciences, University of Rijeka [640-01/20-01/71]. All participants gave written informed consent by filling in an electronic form.

### Materials

The experiment was built using jsPsych ( de Leeuw *et al.*, 2023) and the jspsych-psychophysics plugin ( Kuroki, 2021). The information sheets and debrief sections of the experiment were built using surveyJS ( https://github.com/surveyjs) and custom JavaScript.

Images of three male and three female faces from the Chicago Face Database ( Ma *et al.*, 2015) were selected. Potential directional cues elicited by peripheral facial features or hair were removed by masking the entire image aside from a central oval area and turning the image to grayscale. Iris position and the lightness of the sclera was manipulated by modifying the original images using GIMP (GIMP version 2.10; https://www.gimp.org/) to create 3 images – where the face appeared to look straight ahead, look to the left and look to the right. The images were presented with a width of 180px.

 The letters presented were consonants that appear with medium to high frequency in English (B, C, D, F, G, H, K, L, M, N, P, R, S, T; previously used by Yeaton & Grainger, 2022). Letters were always capitalized and presented in ‘Open Sans’ font with a font size of 48px and a font weight of 800.

 All stimuli were presented within a HTML5 canvas element 800px by 450px. The code used to create the experiment can be found at https://github.com/elegg/gaze-cueing-wm-verbal-experiment. The experiment itself was deployed using JATOS ( Lange *et al.*, 2015) and hosted at https://mindprobe.eu/.

### Procedure

**Figure 1.**
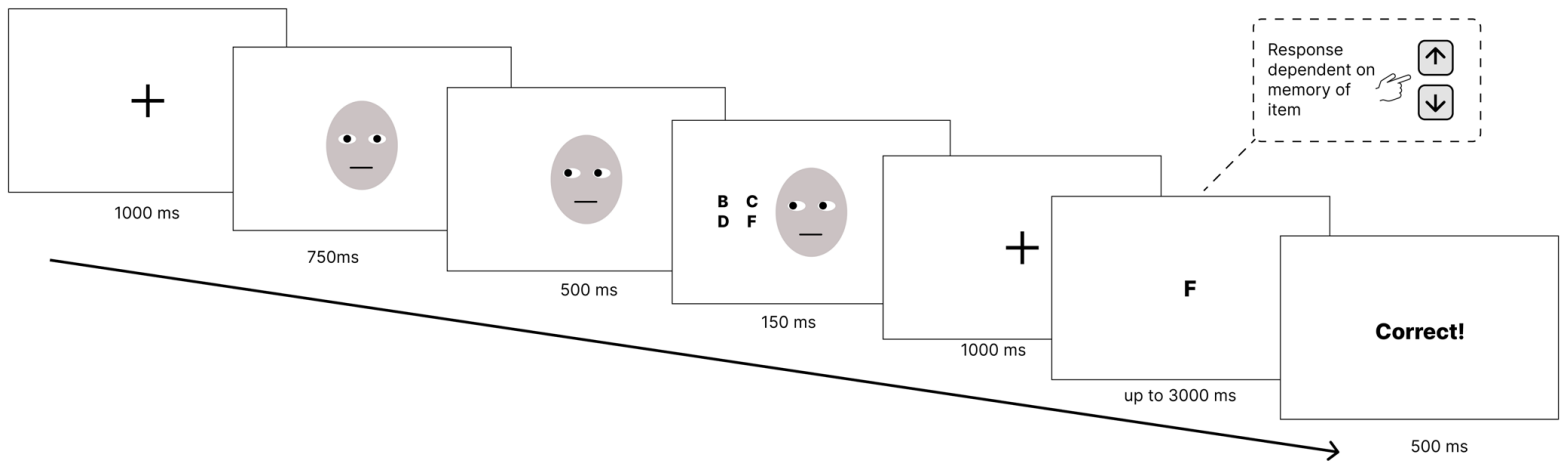
Example trial sequence. *Note:* An example of a trial. First a fixation cross is shown, then a face with eyes facing directly at the participant. The eyes on the face are then shown looking to the left or right and after a 500 ms stimulus onset asynchrony a grid of 4 letters appeared for 150 ms. This was followed by a 1000 ms retention interval before a probe stimulus appeared for up to 3000 ms or until the participant made a response. Participants then received feedback based on their response. *Please note that due to restrictions on distributing images from the Chicago Face Database ( Ma *et al.*, 2015) a schematic image of a face has been used in place of the images used in the experiment.*

At the start of each trial, a central black fixation cross was displayed for 1000 ms. This was followed by a face with direct gaze (central iris position) shown for 750 ms. An image of the same face with the position of the irises in each eye shifted either to the left or right of their previous position - to indicate a change in gaze direction - was presented for 500 ms in the absence of any other stimuli. This image continued to be displayed for a further 150 ms ^1^ during which a 2x2 grid of letters appeared to either the left or the right of the face. Next, a central black fixation cross was displayed for 1000 ms. The display of this fixation cross acted as the retention interval before a single black letter was presented in the centre of the screen. This letter was presented for a maximum of 3000 ms or until participants made a response. A response was made either using the up-arrow key to indicate that the letter was an item from the previously presented letter grid or the down-arrow key to indicate that the letter was absent from the grid. Participants then received feedback for 500 ms depending on whether they had answered correctly, incorrectly or had not made a response within 3000 ms (see Figure 1).

Participants first received 12 practice trials. They then received a total of 196 trials divided into 4 blocks of 48 trials. In half of the trials, the letter grid was presented in the same direction that the eyes on the central face looked toward ( *gazed at* condition; ‘valid condition’ sensu Gregory & Jackson, 2017) and in the other half, the letter grid was presented in the other direction ( *gazed away* condition; ‘invalid condition’ sensu Gregory & Jackson, 2017).

### Analysis

Our primary measure of interest was the discriminability index d’ as an indicator how well participants could detect whether the item presented at test was one of the items they had previously been shown based on both hits (correct detections) and misses (false detections). We calculated d’ as

                                                
*Z(proportion of correct detections) – Z(proportion of false detections)*

For each participant, we calculated d’ For all trial types together as well as separately for trials in the *gazed at* condition and for trials in the *gazed away* condition. Following Gregory and Jackson (2017), we excluded any participants whose d’ score was not within 2.5 mean absolute deviations (see Leys *et al.*, 2013 for an explanation of the benefits of using mean absolute deviation to remove outliers) of the median. This led to data from one participant being excluded from the final analysis. A paired sample t-test was used to compare participants’ d’ scores between the *gazed at* condition and the *gazed away* condition.

We also analysed participants’ response times. For this analysis, we first removed all trials that had incorrect responses. For each participant, we calculated their median overall response time and their median response time for the *gazed at* condition and the *gazed away* condition. Data from participants whose median response time was not within 2.5 mean absolute deviations of the pooled median were excluded. This led to data from one participant being excluded from the final analysis. A paired sample t-test was used to compare participants’ median response times between the *gazed at* condition and the *gazed away* condition.

## Results

### Accuracy - d’

**Figure 2.**
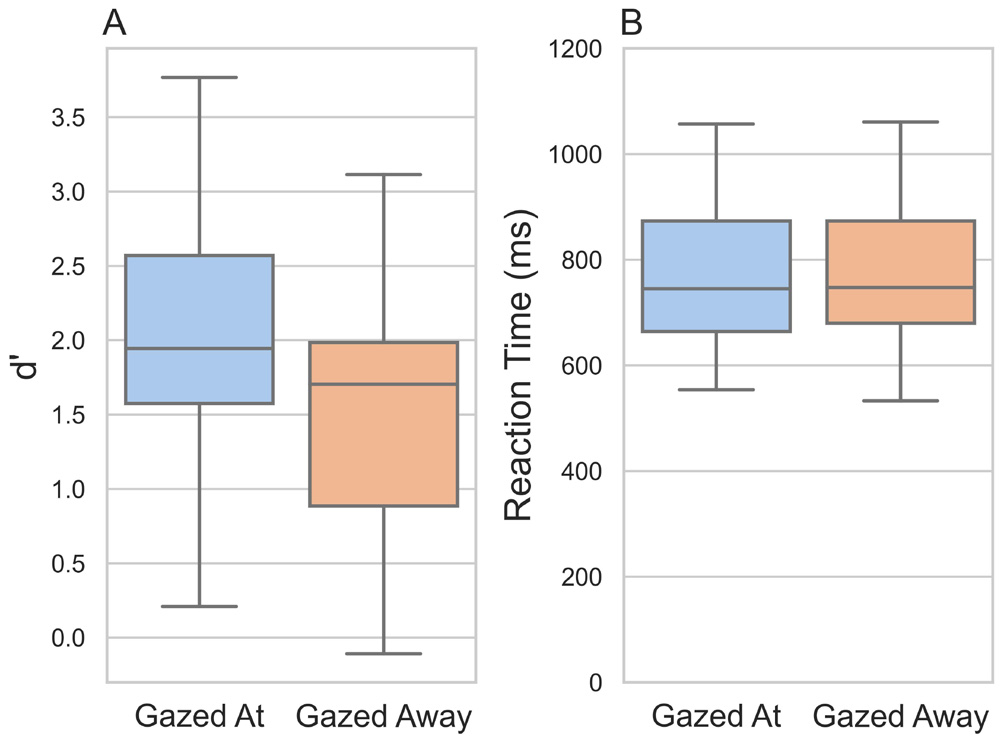
Discriminability index d’ and reaction times in the *gazed at* and *gazed away* conditions. *Note*: **A**) Box and whisker plots showing median and interquartile range of d’ for the *gazed at* and *gazed away* conditions. **B**) Box and whisker plots showing median and interquartile range of participants’ reaction times on correctly answered trials for the *gazed at* and *gazed away* conditions.

Participants were better able to detect whether they had seen the letter before in the *gazed at* condition (M = 2.04, SD = 0.79) than in the *gazed away* condition (M = 1.58, SD = 0.0.78; t(33) = 5.72, *p* <001, d = 0.58; Figure 2A).

### Reaction times

We did not detect a statistically significant difference in the time it took participants to respond between the two conditions (t(33) = 0.967, *p =*.34, d = 0.05; *gazed at* condition: M = 770.00 ms, SD = 131.00 ms; *gazed away* condition: M = 762.00 ms, SD = 133.00 ms; Figure 2B).

## Discussion

Our results showed that participants were more accurate in judging whether they had previously seen a letter when - at the time it was presented - another person appeared to be looking at it ( *gazed at* condition) than when another person appeared to be looking away from it ( *gazed away* condition). Further, we found no significant difference in the time it took for participants to respond to the probe trials between these two conditions, suggesting that the improvements in participants’ memory of letters are not explained by participants trading their accuracy for speed in the *gazed at* condition. Thus, in terms of the influence of gaze cues on working memory, the current results act as a conceptual replication of Gregory and Jackson (2017), who showed that joint attention improves working memory for non-verbalizable content such as colour, and suggest that this improvement is also possible for verbalizable content such as letter.

The effect of the gaze cue on participants’ memory closely resembles the results found by Gregory and Jackson (2017) but our reaction time results do not. Whereas Gregory and Jackson (2017) found evidence that participants were faster to respond to the probe in gazed at trials than gazed away trials, we found no evidence for such an effect. The absence of this effect in the current study may be the result of the change in the type of stimulus between the two studies. Alternatively, it is possible that this effect on reaction times is only observed when working memory load is high and is thus not observable in our study – in the current study, we used only 4 to-be-remembered items whereas the original study used 4, 6 and 8 items. However, the most plausible explanation is that this may be a weak effect that was not found in the current study due to lower statistical power of the statistical test than needed to detect such an effect – an interpretation supported by the failure of other follow-up studies to reliably detect such an effect on reaction times Gregory & Jackson, 2019). The current study was powered to detect a mid-sized effect, as expected for an effect of the gaze cue on the main measure of interest (d’). Critically, the effect of the gaze cue on participants’ reaction times to a probe stimulus has limited bearing on our main question of how such gaze cues influence memory. Here, and in previous studies, the primary concern with reaction times was that participants may trade speed for accuracy such that their relatively inaccurate responding in the gazed away condition would be explained by them responding very fast. The absence of any such pattern of results in the current study and the inverse effect found by Gregory and Jackson (2017) suggest that such a trade-off is unlikely to explain the observed difference in memory accuracy. Thus, our findings extend those by Gregory and Jackson (2017) and suggest that joint attention can improve working memory for both verbalizable and non-verbalizable content, which are thought to be processed separately ( Baddeley, 2003).

To conclude, our findings suggest that joint attention can improve working memory for verbalizable content such as letters. As such, the current results help connect two previous studies, specifically Dodd *et al.* (2012) who demonstrated that joint attention improves long-term memory for verbalizable stimuli - words - and Gregory and Jackson (2017) who demonstrated that joint attention improves working memory for visual stimuli – colour. Thus, our study presents an important step towards showing that joint attention can modulate different types of memory for various types of stimuli that may be represented by different subsystems.

## Ethics and consent

The study was approved by the Ethics Committee for Research at the Faculty of Humanities and Social Sciences, University of Rijeka [640-01/20-01/71].

All participants gave informed written consent to participate by filling in an online (electronic) consent form.

## Data Availability

The raw data from certain trials of the experiment contain personal identifiable information and cannot be made publicly available due to ethical considerations. However, the following data has been made available and contains all information necessary to replicate our analysis, results and data visualisations. Zenodo: Data From "Gaze improves working memory for verbal items", https://doi.org/10.5281/zenodo.17491711 ( Legg *et al.*, 2025a) This project contains the following data: “gaze-working-memory-data.csv” which contains the trial by trial data for each participantData are available under the terms of the Creative Commons Attribution 4.0 International license (CC-BY 4.0) Data are available under the terms of the Creative Commons Attribution 4.0 International license (CC-BY 4.0)

## Software availability statement

Software used in the experiment is available from:

- Source code available from: https://github.com/elegg/gaze-cueing-wm-verbal-experiment
- Archived software: https://doi.org/10.5281/zenodo.17426209
- License: MIT License

Software used for analysis and visualisations is available from:

- Source code available from: https://github.com/elegg/gaze-cueing-memory-verbal-analysis
- Archived software available from: https://doi.org/10.5281/zenodo.17426275
- License: MIT License

### Extended data

Zenodo: Extended Data From "Gaze improves working memory for verbal items",

https://doi.org/10.5281/zenodo.17549330 ( Legg *et al.*, 2025b)

This project contains the following data:

• Extended Data - Gaze-WM.docx (a word document containing: 1. an example consent form questionnaire, 2. an example of the instructions – text and image - presented to participants, and 3. an example of the text presented to the participant in the debrief section of the experiment).
  Data are available under the terms of the Creative Commons Attribution 4.0 International license (CC-BY 4.0)
