# Gaze cues and arrow cues modulate accuracy of recall of verbal items in a working memory task

**DOI:** 10.12688/openreseurope.21720.2

**Published:** 2026-02-26

**Authors:** Vinoprasath Shivakumar, Ljerka Ostojić, Edward Legg

**Affiliations:** 1 Division of Cognitive Sciences, Faculty of Humanities and Social Sciences, University of Rijeka, Rijeka, Primorje-Gorski Kotar, 51000, Croatia; 2 Department of Psychology, Faculty of Humanities and Social Sciences, University of Rijeka, Rijeka, Primorje-Gorski Kotar, 51000, Croatia; 3 Centre For Mind and Behaviour, University of Rijeka, Rijeka, Primorje-Gorski Kotar County, 51000, Croatia

**Keywords:** gaze cueing, working memory, joint attention, attention orienting

## Abstract

**Background:**

Previous studies have shown that items that another individual looks at are better remembered than items that are not looked at, whereas the same effect has rarely been observed for non-social cues such as arrows. This pattern of results has been taken as an indication that joint attention improves memory. However, these previous studies have differed in the type of memory being tested and the type of content that is to be remembered: while effects of joint attention on long-term memory were tested with verbal items and non-verbal items, effects on working memory have only been tested with non-verbal items such as colour. Thus, the aim of the current study was to extend these previous findings and investigate whether joint attention influences working memory for verbal items.

**Methods:**

In Experiment 1, participants were first presented with an image of a face with eyes that gazed either to the left or to the right. A grid of 4 letters (2x2) was then shown either on the side cued by gaze or on the opposite side. After a retention interval (1000 ms), participants were shown a letter in the centre of the screen and judged whether this letter was part of the grid shown before. In Experiment 2, we followed the same general procedure but one group was presented with gaze cues, and the other group was presented with arrow cues.

**Results:**

Across two experiments, our results revealed that participants had better recall of letters that had been cued than letters that had not been cued, regardless of cue type. In contrast, participants’ reaction times were not influenced by whether the letter had been cued.

**Conclusions:**

Our findings suggest that both social and non-social cues can modulate recall of verbal items such as letters in a working memory task.

## Introduction

The physiology of human eyes with their large white sclera and dark iris helps provide a clear signal of the direction in which an individual is looking ( Kobayashi & Kohshima, 1997). A large body of research using manipulations to face and eye images that reflect shifts in the iris position relative to the sclera have demonstrated that humans are remarkably sensitive to the direction of another’s gaze. Already 3-month old infants shift their own attention in the direction signalled by images of eyes looking to the left or right placed on an image of a face ( Hood *et al.*, 1998). For adults, such shifts in attention appear to have some degree of automatization, appearing in situations where gaze fails to predict a target’s location and when participants are placed under cognitive load ( Friesen & Kingstone, 1998; Visser & Roberts, 2018).

This sensitivity to the direction of another’s gaze further allows two individuals to coordinate their attention toward the same external stimulus. The benefits of such ‘joint attention’ have been the subject of extensive discussion ( Kwisthout *et al.*, 2008; Moore *et al.*, 2014; Tomasello, 1995). One strand of research on the downstream consequences of joint attention focuses on processes that have played key roles in the discussion about the evolution of joint attention – such as the impact of joint attention on word learning ( Hirotani *et al.*, 2009) and on conveying information about preferences ( Bayliss *et al.*, 2006; Bayliss *et al.*, 2007). A second strand of research investigates the impact of joint attention on domain-general processes that could be affected by an individual’s previous level of attention, such as working memory ( Gregory & Jackson, 2017, 2019) and long-term memory ( Dodd *et al.*, 2012; Ishikawa & Yoshioka, 2025).

Dodd *et al.* (2012) demonstrated the effect of gaze cues on recall of items stored in long-term memory. Participants first saw a schematic face that did not have pupils/irises. Then, the pupils/irises were shown, making the face appear to look to the left or to the right. Finally, a word was shown either to the left or to the right side of the face, such that the word appeared either on the side that the eyes were looking toward (cued words) or on the opposite side (uncued words). When asked to recall the words at the end of the experiment, participants remembered words better when they had appeared in the location that the central face had been looking at, suggesting that gaze cues may improve long-term memory of written words. Complementing these results, Gregory and Jackson (2017) found that participants showed higher accuracy of recall for cued items than for uncued items in a working memory task. Here, participants were presented with a grid of 4, 6 or 8 coloured squares either on the side that a central face gazed toward or on the opposite side. There were thus two critical differences between these two studies. First, in Dodd *et al.* (2012), the to-be-remembered items were words, and in Gregory and Jackson (2017), they were square patches of colour – such that in the former, the memory test was based on verbal information, and in the latter, the memory test was based on non-verbal information. Second, in Dodd *et al.* (2012), the test was conducted at the end of the presentation of all to-be-remembered items, and in Gregory and Jackson (2017), the test was conducted 1000 ms after the offset of the to-be-remembered items – such that in the former, accuracy in recalling items stored in long-term memory was assessed, and in the latter, accuracy in recalling items in working memory was assessed. Critically - based on a wider debate within the gaze cueing literature regarding the specificity of the underlying mechanism ( Capozzi & Ristic, 2020; Chacón-Candia *et al.*, 2023; Hietanen *et al.*, 2008; Marotta *et al.*, 2012; Ristic *et al.*, 2002) - both studies also tested whether a non-social stimulus that can orient attention, namely an arrow cue, would improve participants’ recall (Experiment 4 in Dodd *et al.*, 2012; Experiment 2 in Gregory & Jackson, 2017). In both cases, the accuracy of recall was not affected, suggesting that the effect on recall may be specific to social cues such as gaze.

Following these two original studies investigating cueing effects on recall of items in long-term memory and in working memory, several follow-up studies have extended the initial findings and some of them employed further tests of the specificity of the gaze cueing effect. For recall of items stored in long-term memory, apart from Dodd *et al.* (2012), only one study investigated the effects of cueing. Ishikawa and Yoshioka (2025) tested 7- to 10-year-old children following a similar procedure to Dodd *et al.* (2012) using fractal patterns instead of words as the to-be-remembered items. Like in Dodd *et al.* (2012), here too, gaze cues, but not arrow cues improved recall of patterns, suggesting that gaze cues also improve recall of *non-verbal* content stored in long-term memory. For items in working memory, for which more studies have been conducted, the results of purely behavioural measures are more mixed. One recent study that only used gaze cues failed to replicate the cueing effect on recall (Experiment 1: Foglino & Wykowska, 2025). Most other studies reported cueing effects on recall that were specific to the gaze cue or only tested the effect of gaze on recall ( Gregory & Jackson, 2019; Gregory & Kessler, 2022). Additionally, in the original study by Gregory and Jackson (2017), one experiment used a motion cue (a vertical line that shifts in space along a horizontal line) as the non-social cue, thus showing that the lack of an effect on recall was not specific to the use of an arrow as the non-social cue. In contrast, a study that used a 3D environment found that recall of information about an object was improved both by a gaze cue and a moving non-social cue ( Gregory *et al.*, 2022). Critically, however, the authors report a difference in the change in theta power between cued and uncued items at the time in which the items were encoded between the two cue types. This was interpreted as evidence that the cueing effects observed at the behavioural level may be differently implemented at the neural level. Taken together, for both long-term memory and working memory, the majority of empirical findings thus support the notion that gaze cues have a specialized role in improving recall.

This notion of a specialized effect of gaze cues on recall is strengthened by results indicating that participants also process information that is unique to social agents. Gregory and Jackson (2019) demonstrated that the effect of gaze was modulated by whether the depicted eyes could see the target. Here, barriers that prevented sight - placed between the gaze cue and the target location - abolished the influence of gaze cues on recall. However, the effect of gaze on recall of items in working memory was found when the barriers had a window in them which allowed the target to be seen from the position of the gaze cue. Furthermore, due to modulation of memory being related to what can be seen from the position of the gaze cues, the authors concluded that this effect may be driven by mentalising (for a review of the links between gaze cueing and mentalising, see Capozzi & Ristic, 2020).

This hypothesized specialized role of gaze cues in improving recall of cued items stands in contrast to results of standard detection tasks, in which both gaze cues and arrow cues orient attention towards a specific location such that participants are faster at responding to cued vs. uncued items (for a meta-analysis, see Chacón-Candia *et al.*, 2023). The results from such detection tasks thus suggest that there is no notable difference in the amount of spatial attention that is being affected by social and non-social cues even if the exact mechanisms underpinning the effects may differ (e.g., Galfano *et al.*, 2012; Ristic *et al.*, 2002). Consequently, because the effect appears to be independent of the influence that gaze and arrows have on attention, most authors interpret the effect of gaze cues on recall as a ‘memory effect’. This interpretation appears to be in line with findings from studies where gaze cues were presented not before but *after* the to-be-remembered item (in this case, a complex polygon shape) was presented and thus encoded in working memory ( Nie *et al.*, 2018; Zhang *et al.*, 2023). These studies found that retroactive gaze cues, but not retroactive motion cues improve recollection – findings that cannot be explained by changes in external spatial attention because the to-be-remembered items are no longer visible.

Before all these results from studies on long-term memory and working memory can be taken as an indication that orienting cues (whether social orienting cues like gaze specifically, or orienting cues more generally) produce improvements to working memory for different types of memory across different stimuli, it is crucial to consider that processes involved in storing verbal and non-verbal information in working memory are commonly proposed to be separate ( Baddeley, 2003). Verbal information is likely being stored within the phonological loop, with information decaying rapidly unless subject to a maintenance process such as subvocal rehearsal ( Camos *et al.*, 2009; although see Oberauer, 2019). In contrast, non-verbal information is thought to be stored in the visuo-spatial sketchpad and is also maintained through maintenance strategies that may involve allocating attention – in the absence of the stimulus – to the spatial location of stimuli that need to be remembered ( Awh *et al.*, 2006). Considering the results of research into the effects of orienting cues on recall, this raises the question of whether for verbal content, orienting cues improve recall only for items stored in long-term memory or whether the improvement is also seen for items in working memory. We investigated this question across two experiments using a modified procedure of Gregory and Jackson (2017). In Experiment 1 we tested whether accuracy of recall of verbal content in working memory is improved by a gaze cue. In Experiment 2 we tested whether this improvement in recall of verbal content was specific to a social cue (gaze cue) or whether it would also occur for a non-social cue (arrow cue).

***Experiment 1***

## Methods

### Participants

A power analysis using the R package ‘pwrrs’ indicated that a sample size of 34 was needed to detect a medium sized effect (cohen’s d = 0.5) with a power of 0.8 using a paired sample t-test with the significance level set at .05. Thirty-six participants with a mean age of 38 (SD = 11.4, range = [19-60]) years were recruited using prolific.com and were compensated £2.25 for taking part in the study. Data files were available for all participants, but two participants were excluded due to deviations in their reaction time or accuracy data (see analysis section). Thus, the final sample was 34 participants. The study was approved by the Ethics Committee for Research at the Faculty of Humanities and Social Sciences, University of Rijeka [640-01/20-01/71]. All participants gave written informed consent by filling in an electronic form.

### Materials

The experiment was built using jsPsych ( de Leeuw *et al.*, 2023) and the jspsych-psychophysics plugin ( Kuroki, 2021). The information sheets and debrief sections of the experiment were built using surveyJS ( https://github.com/surveyjs) and custom JavaScript.

Images of three male and three female faces from the Chicago Face Database ( Ma *et al.*, 2015) were selected. Potential directional cues elicited by peripheral facial features or hair were removed by masking the entire image aside from a central oval area and turning the image to grayscale. Iris position and the lightness of the sclera was manipulated by modifying the original images using GIMP (GIMP version 2.10; https://www.gimp.org/) to create 3 images – where the face appeared to look straight ahead, look to the left and look to the right. The images were presented with a width of 180px.

The letters presented were consonants that appear with medium to high frequency in English (B, C, D, F, G, H, K, L, M, N, P, R, S, T; previously used by Yeaton & Grainger, 2022). Letters were always capitalized and presented in ‘Open Sans’ font with a font size of 48px and a font weight of 800.

All stimuli were presented within a HTML5 canvas element 800px by 450px. The code used to create the experiment can be found at https://github.com/elegg/gaze-cueing-wm-verbal-experiment. The experiment itself was deployed using JATOS ( Lange *et al.*, 2015) and hosted at https://mindprobe.eu/.

### Procedure

**Figure 1.**
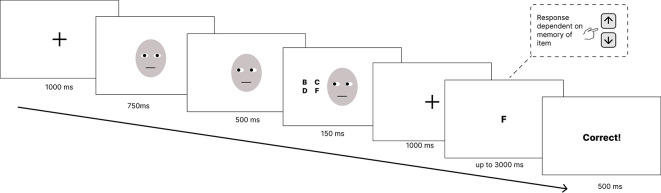
Example trial sequence. *Note:* An example of a trial. First a fixation cross is shown, then a face with eyes facing directly at the participant. The eyes on the face are then shown looking to the left or right and after a 500 ms stimulus onset asynchrony a grid of 4 letters appeared for 150 ms. This was followed by a 1000 ms retention interval before a probe item appeared for up to 3000 ms or until the participant made a response. Participants then received feedback based on their response. *Please note that due to restrictions on distributing images from the Chicago Face Database (* *Ma et al., 2015* *) a schematic image of a face has been used in place of the images used in the experiment.*

At the start of each trial, a central black fixation cross was displayed for 1000 ms. This was followed by a face with direct gaze (central iris position) shown for 750 ms. An image of the same face with the position of the irises in each eye shifted either to the left or right of their previous position - to indicate a change in gaze direction - was presented for 500 ms in the absence of any other stimuli. This image continued to be displayed for a further 150 ms ^1^ during which a 2x2 grid of letters appeared to either the left or the right of the face. Next, a central black fixation cross was displayed for 1000 ms. The display of this fixation cross acted as the retention interval before a single black letter (probe item) was presented in the centre of the screen. This letter was presented for a maximum of 3000 ms or until participants made a response. A response was made either using the up-arrow key to indicate that the letter had been present in the previously presented letter grid or the down-arrow key to indicate that the letter had been absent from the grid. Participants then received feedback for 500 ms depending on whether they had answered correctly, incorrectly or had not made a response within 3000 ms (see Figure 1).

Participants first received 12 practice trials. They then received a total of 192 trials divided into 4 blocks of 48 trials. In half of the trials, the letter grid was presented in the same direction that the eyes on the central face looked toward ( *cued* condition; ‘valid condition’ sensu Gregory & Jackson, 2017) and in the other half, the letter grid was presented in the other direction ( *uncued* condition; ‘invalid condition’ sensu Gregory & Jackson, 2017). Further, in half of trials the probe item judged by participants had been present in the letter grid. Forty-eight trials were used per block because this reflects all possible combinations of 6 faces, 2 gaze directions, 2 locations for the letter grid and 2 options for whether the probe item had been presented or not. The grid of letters was constructed for every trial by randomly sampling (without replacement) from the 14 consonants. On the 50% of trials where the probe had been present in the grid, one of the four letters was randomly selected to be the probe. In the other 50% of trials, a fifth letter was sampled from the remaining consonants. The order of these 48 trials was randomised within each of the 4 blocks.

### Analysis

Our primary measure of interest was the discriminability index d’ as an indicator how well participants could detect whether the item presented at test (probe item) was one of the items they had previously been shown based on both hits (correct detections) and misses (false detections). We calculated d’ as

*Z (proportion of correct detections) – Z (proportion of false detections)*

For each participant, we calculated d’ for all trial types together as well as separately for trials in the *cued* condition and for trials in the *uncued* condition. Following Gregory and Jackson (2017), we excluded any participants whose d’ score was not within 2.5 median absolute deviations (see Leys *et al.*, 2013 for an explanation of the benefits of using median absolute deviation to remove outliers) of the pooled median. This led to data from one participant being excluded from the final analysis. A paired sample t-test was used to compare participants’ d’ scores between the *cued* condition and the *uncued* condition.

For a secondary measure, we also analysed participants’ response times. This was done primarily to ensure that any potential difference in the participants’ accuracy between the two conditions was not due to a speed-accuracy trade off. For this analysis, we first removed all trials that had incorrect responses. For each participant, we calculated their median overall response time and their median response time for the *cued* condition and the *uncued* condition. Data from participants whose median response time was not within 2.5 median absolute deviations of the pooled median were excluded. This led to data from one participant being excluded from the final analysis. A paired sample t-test was used to compare participants’ median response times between the *cued* condition and the *uncued* condition.

After exclusion of participants based on d’ scores and reaction times, the final sample size for all analyses was 34.

## Results

### Accuracy - d’

**Figure 2.**
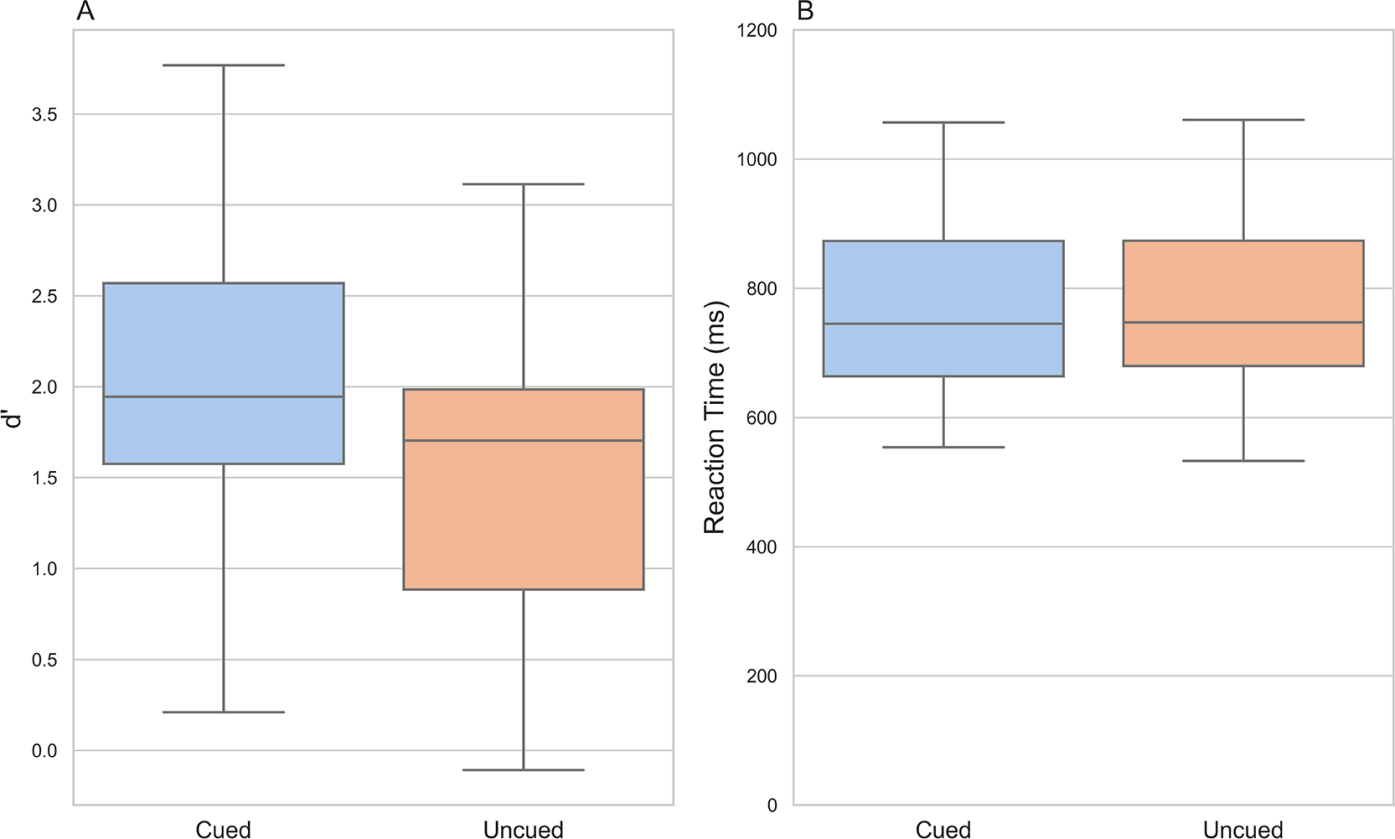
Discriminability index d’ and reaction times in the *cued* and conditions. *Note*: **A**) Box and whisker plots showing median and interquartile range of d’ for the *cued* and *uncued* conditions. **B**) Box and whisker plots showing median and interquartile range of participants’ reaction times on correctly answered trials for the *cued* and *uncued* conditions.

Participants were better able to detect whether they had seen the letter before in the *cued* condition (M = 2.04, SD = 0.79) than in the *uncued* condition (M = 1.58, SD = 0.0.78; t(33) = 5.72, *p* <001, d = 0.58; Figure 2A).

### Reaction times

We did not detect a statistically significant difference in the time it took participants to respond between the two conditions (t(33) = 0.967, *p =.*34, d = 0.05; *cued* condition: M = 770.00 ms, SD = 131.00 ms; *uncued* condition: M = 762.00 ms, SD = 133.00 ms; Figure 2B).

## Discussion

Our main results in Experiment 1 showed that participants were more accurate in judging whether they had previously seen a letter when - at the time it was presented - a gaze cue was directed towards it ( *cued* condition) than when the gaze cue was directed away from it *(uncued* condition). Thus, the results regarding the effect of gaze cueing on participants’ recall of letters found in our study closely resembles the results found by Gregory and Jackson (2017) for colour squares.

As a secondary result, we found no statistically significant effect of gaze cueing on the time it took participants to respond to whether they had previously seen the probe item. This contrasts with the findings by Gregory and Jackson (2017), where participants were not just more accurate but also faster to respond to the probe item when it had been cued. This divergence in findings may be explained by the differences in the type of the to-be-remembered content (verbal content in our study and non-verbal content in Gregory and Jackson (2017)) or differences in the working memory load (our study used only 4 to-be-remembered items, whereas Gregory and Jackson (2017) used 4, 6, and 8 items). The most plausible explanation, however, is that this effect is weak and not found in the current study due to lower statistical power of the statistical test than needed to detect such an effect. The current study was powered to detect a mid-sized effect, as expected for an effect of the gaze cue on the main measure of interest (d’). This interpretation is further supported by the lack of an effect on reaction times in other follow-up studies to the original Gregory and Jackson (2017) study (e.g., Gregory & Jackson, 2019). Critically, the effect of the gaze cue on participants’ reaction times to a probe item has limited bearing on our main question of whether gaze cues influence working memory. This is because here, like in previous studies, the primary concern with reaction times is that participants may trade speed for accuracy. The absence of any such pattern of results in the current study and the inverse effect found by Gregory and Jackson (2017) suggest that such a trade-off is unlikely to explain the observed difference in recall accuracy.

In conclusion, the findings of Experiment 1 suggest that gaze cues may not only increase recall of non-verbal content ( Gregory & Jackson, 2017) but also of verbal content. However, in light of the debate about whether such an improvement of recall may be based on spatial attention or is directly related to changes in working memory itself and in light of previous studies highlighting that the effect for non-verbal items may be specific to social, gaze cues, ( Dodd *et al.*, 2012; Gregory & Jackson, 2017, 2019; Ishikawa & Yoshioka, 2025) it was necessary to test to what extent the effect observed in Experiment 1 was specific to the gaze cue. In Experiment 2, we thus tested two groups of participants, one in which the cue was social (gaze cue) – this group thus acted as a direct replication of Experiment 1 – and one in which the cue was non-social (arrow cue).

***Experiment 2***

## Methods

### Participants

A power analysis using the R package ‘pwrrs’ indicated that a sample size of 152 was needed to detect the estimated effect size of partial η ^2^ of 0.051 with a power of 0.80 using a 2x2 ANOVA with one between-subject and one within-subject factor. The estimated effect size was based on the size of the interaction found by Gregory and Jackson (2019) between barrier type (transparent vs. opaque) and consistency. This size of the cueing effect is substantially smaller than the effect size found for the interaction between cue type and consistency by Ishikawa & Yoshioka (2025) partial η ^2^ of 0.366. A total of 168 participants with a mean age of 41.4 (SD = 10.7, range= [20-60]) years were recruited using prolific.com and were compensated £2.25 for taking part in the study. Data files were missing from two participants, and nine participants were excluded due to deviations in their reaction time or accuracy data (see analysis section). Thus, the final sample was 157 participants. The study was approved by the Ethics Committee for Research at the Faculty of Humanities and Social Sciences, University of Rijeka [640-01/20-01/71]. All participants gave written informed consent by filling in an electronic form.

### Materials

**Figure 3.**
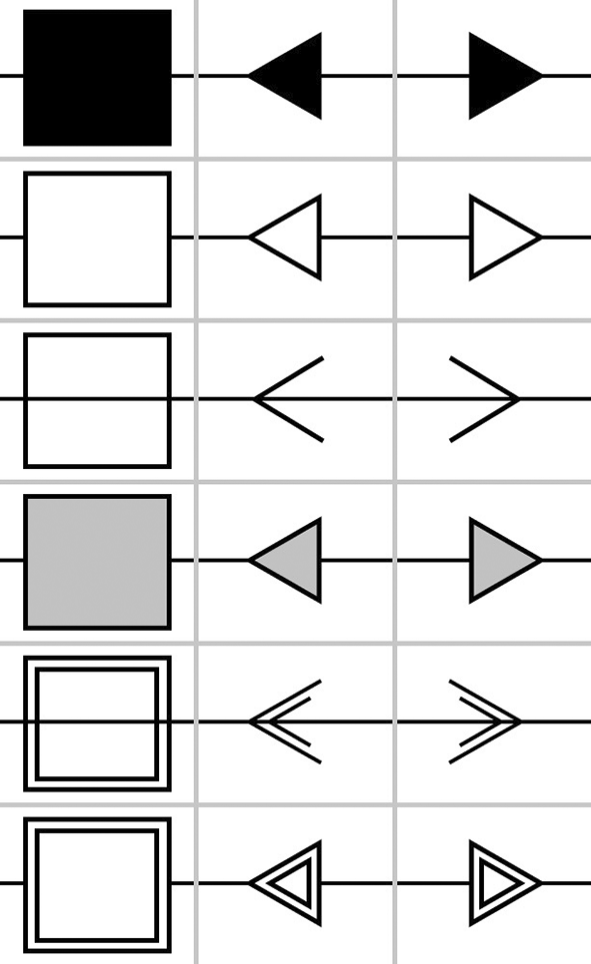
Arrow Exemplars. *Note:* The six exemplars of arrows presented during the study as non-social cues. The square in the left column represents the non-directional image, the central column represents the left pointing arrows and the right column the right pointing arrows.

The experiment was built using the same procedure as in Experiment 1. The same 6 face stimuli used in Experiment 1 were used as orienting cues for the gaze version of the task. For the arrow version of the task, six different arrows in different styles were created using Figma ( figma.com; see Figure 3) to provide the non-social orienting cues. In addition, six squares in corresponding styles to the arrows were used to provide non-directional stimuli for the non-social cues equivalent to the initial presentation of the face with direct gaze. The letters presented were the same and followed the same procedure as in Experiment 1.

**Figure 4.**
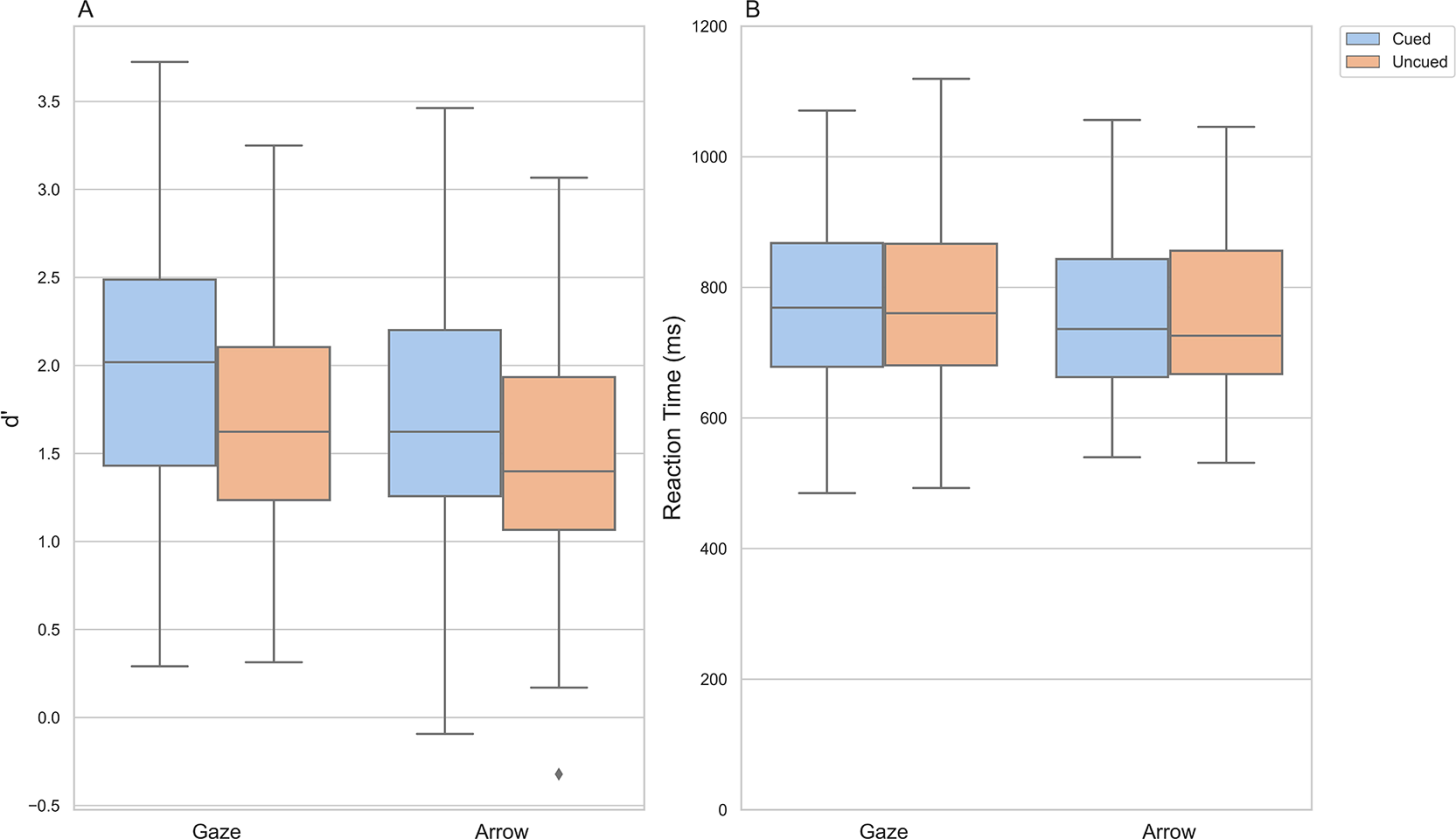
Discriminability index d’ and reaction times in the *cued* and *uncued* conditions. *Note*: **A**) Box and whisker plots showing median and interquartile range of d’ for the *cued* and *uncued* conditions. **B**) Box and whisker plots showing median and interquartile range of participants’ reaction times on correctly answered trials for the *cued* and *uncued* conditions.

Like in Experiment 1, all stimuli were presented within a HTML5 canvas element 800px by 450px. The code used to create the experiment can be found at https://github.com/elegg/gaze-cueing-wm-verbal-experiment. The experiment itself was deployed using JATOS ( Lange *et al.*, 2015) and hosted at https://mindprobe.eu/.

### Procedure

The procedure was the same as in Experiment 1 except that at the start of testing, participants were assigned to either the gaze or arrow version of the task. Thus, participants only received instructions and test trials relating to one type of cue. Participant allocation was assigned such that the two groups were filled as close to equal as possible. If, at the time of starting the experiment, fewer participants had been assigned to one group, then the participant was assigned to the other group. If the same number of participants had been assigned to each group, then participants were assigned to one of the two groups with a probability of .5.

### Analysis

We calculated d’ for each participant as described in Experiment 1. Further, we followed the same procedure for excluding participants as in Experiment 1 (exclusion if d’ score was not within 2.5 median absolute deviations of the pooled median). This led to 4 exclusions. A 2 (cue type: gaze vs. arrow) x 2 (consistency: cued vs. uncued) ANOVA was used to analyse d’ scores. In addition, for each cue type, we conducted a paired t-test to compare participants’ d’ in the *cued* and *uncued* conditions.

Participants’ median response times were calculated as described in Experiment 1. Again, we used the same exclusion criteria as in Experiment 1 (exclusion if median response time was not within 2.5 median absolute deviations of the pooled median). This led to 5 exclusions. A 2 (cue type: gaze vs. arrow) x 2 (consistency: cued vs. uncued) ANOVA was used to analyse reaction times.

After exclusion of participants based on availability of data files, d’ scores, and reaction times, the final sample size for all analyses was n = 157.

## Results

### Accuracy - d’

For both types of cue, participants were better able to detect whether they had seen the letter when the letters had been cued than when they had not been cued (arrow - cued: M =1.71, SD = 0.79; arrow - uncued: M = 1.49, SD = 0.66; gaze - cued: M = 1.93, SD = 0.75, gaze - uncued: M = 1.62, SD = 0.66). This was observed as a statistically significant difference between the conditions for each of the cue types separately (arrow: t(78) = 4.08, p < .001, d = 0.30; gaze: t(77) = 4.56, *p* < .001, d = 0.44) and as a statistically significant main effect of consistency in a two-way ANOVA (F(1, 155) = 37.405, *p* < .001, η _p_^2^ = 0.194). The ANOVA revealed no statistically significant main effect of cue type (F(1, 155) = 2.522, p = .31, η _p_^2^ = 0.016) and no statistically significant interaction between cue type and consistency (F(1, 155) = 1.061, *p* = .305, η _p_^2^ = 0.007).

### Response times

The two-way ANOVA revealed no statistically significant main effect of cue type (F(1, 155) = 0.427, *p* = .515, η _p_^2^ = 0.003) and no statistically significant main effect of consistency (F(1, 155) = 0.659, *p* = .418, η _p_^2^ =0.004) on participants’ reaction times. There was also no statistically significant interaction effect between cue type and consistency on participants’ response times (F(1, 155) = 0.996, *p* = .32, η _p_^2^ =0.006).

## Discussion

The results of Experiment 2 showed that, regardless of cue type, participants were more accurate at judging whether they had previously seen a letter when – at the time it was presented – it had been cued than when it had not been cued. The improved recall of cued letters observed in the group in which the cue was a social stimulus (gaze) thus replicated the findings from Experiment 1. Because this effect was also observed in the group presented with a non-social cue (arrow) and there was no statistically significant difference regarding this effect between the two cue types, our results suggest that the observed effect on recall is not specific to social stimuli such as gaze cues. Like in Experiment 1, we found no significant difference in the time it took participants to respond, regardless of cue type and consistency, strengthening the suggestion that the improved accuracy for cued items cannot be explained by a speed-accuracy trade-off.

### General Discussion

Across two experiments, we demonstrated that orienting cues increase the accuracy of participants’ recall for letters presented at the cued location compared to uncued letters in a working memory task. In Experiment 1, we demonstrated this effect for gaze cues and in Experiment 2, we replicated the effect for gaze cues and demonstrated that the effect also occurs for arrow cues. Notably, we found that there was no statistically significant difference in the effect between gaze and arrow cues. Thus, the results of the two experiments indicate that recall for verbal content in working memory can be improved when the content is presented at a cued location regardless of whether the cue is considered social (gaze) or non-social (arrows). A cue-driven change in spatial attention may provide the most likely and parsimonious explanation for why verbal items that are cued are remembered better than uncued items. When spatial attention is drawn away from the to-be-remembered items (i.e., uncued items), participants need to re-orient towards the location of the to-be-remembered items, such that the length of time these items are attended to (and in which they can be encoded) is less than for cued items for which no re-orientation is needed ( Alister *et al.*, 2024).

The findings of Experiment 2 contrast with the results of the most closely related studies of attentional cueing on working memory ( Gregory and Jackson, 2017, 2019): while the results of Experiment 2 regarding the impact of *gaze cues* act as a conceptual replication of these previous studies, the results regarding the impact of *arrow cues* clearly differ from the results of these previous studies because there, arrow cues produced no improvement in recall. Although the specific effect of gaze cues on recall has also been reported by other studies, Gregory *et al.* (2022) found that both social (head movement) and non-social (movement of a stick) cues improved participants’ ability to recollect information about 3D objects. Consequently, we need to consider what could explain why some studies find that social but not non-social cues influence recall while other studies find an effect on recall also for non-social cues (namely Gregory *et al.*, 2022, and our Experiment 2).

Gregory *et al.* (2022) proposed the increased salience of the moving non-social stimulus as a potential explanation of why they found a cueing effect on recall also with a non-social orienting cue. Relatedly, in Experiment 2 of the current study, in order to be able to make a generalised claim regarding arrows (and not a single arrow) and provide the equivalent number of different arrows to the number of different faces in the gaze cue group, we presented participants in the arrow group with six different arrow exemplars. A consequence of this was that the arrows in the current study may have been more salient to participants than in previous studies, where a single arrow has been used. Critically, these previous studies used multiple exemplars of faces for the social orienting cue but only a single exemplar for the non-social orienting cue ( Gregory & Jackson, 2017, 2019; Ishikawa & Yoshioka, 2025; Nie *et al.*, 2018; Zhang *et al.*, 2023; an exception is Dodd *et al.*, 2012 who used a single schematic face). The use of multiple exemplars may reduce habituation to a repeatedly presented stimulus ( Thompson, 2009). In the case of faces, repetition of the same face is known to reduce typical responses but continuous presentation of different faces does not ( Gauthier *et al.*, 2000). Thus, the difference in the number of arrow exemplars presented in previous studies may have reduced the salience of the arrow cues in a way that the salience of multiple gaze cues did not. Consequently, we cannot rule out that an effect also for non-social cues would be found in studies on recall of non-verbal content in working memory tasks when both social and non-social cues have similar levels of salience.

In addition to highlighting the question about the salience of the orienting cue, our results open up further research avenues and theoretical considerations for cueing effects on memory. In regard to cueing effects on recall in working memory tasks, future work may need to systematically investigate the possibility of an interaction between the type of orienting cue (social vs. non-social) and the nature of the to-be-remembered items (verbal vs. non-verbal). Theoretical implications of such an interaction should consider that spatial attention – to the extent that it is assumed to be similar for both social and non-social orienting cues – cannot provide the full explanation of the effect of cues on recall, because spatial attention would be the same both for verbal and non-verbal to-be-remembered-items. Thus, the divergence of patterns across cues would indicate that the way that verbal and non-verbal content is stored within working memory plays a crucial role in whether a cue will improve recall. In line with this idea that the way that verbal content is stored in working memory matters, a recent review of verbal interference paradigms highlights that covert language tends to play a role in memory when the to-be-remembered items have a readily available label ( Nedergaard *et al.*, 2023). Finally, in regard to cueing effects on long-term memory, it remains to be investigated why for verbal content, both gaze cues and arrow cues are able to produce a cueing effect on recall in a working memory task, while this effect is reported to only carried forward to long-term memory for gaze cues specifically ( Dodd *et al.*, 2012).

## Conclusion

The current study sought to extend previous findings that have demonstrated an impact of social cues on memory. In the case of long-term memory, it has previously been found that a social cue but not a non-social cue (arrows) improves accuracy for recall of both verbal items (words) and non-verbal items (colour). In the case of working-memory, it has been found that a social cue improved accuracy for recollecting non-verbal items. The results of Experiments 1 and 2 indicate that both gaze and arrows modulate recall of verbal items in a working memory task. For these current results, spatial attention appears to be sufficient in explaining why orienting cues modulate recall in a working memory task for verbal content.

## Ethics and consent

The study was approved by the Ethics Committee for Research at the Faculty of Humanities and Social Sciences, University of Rijeka [640-01/20-01/71].

All participants gave informed written consent to participate by filling in an online (electronic) consent form.

## Data Availability

The raw data from certain trials of the experiment contain personal identifiable information and cannot be made publicly available due to ethical considerations. However, the following data has been made available and contains all information necessary to replicate our analysis, results and data visualisations. Zenodo: Data From “Gaze cues and arrow cues modulate accuracy of recall of verbal items in a working memory task”, https://doi.org/10.5281/zenodo.18294236 ( Legg *et al.*, 2026a). This project contains the following data:
• “gaze-working-memory-data.csv” which contains the trial by trial data for each participant from Experiment 1. Data are available under the terms of the Creative Commons Attribution 4.0 International license (CC-BY 4.0) • “gaze-arrow-wm-data.csv” which contains the trial by trial for each participant from Experiment 2. Data are available under the terms of the Creative Commons Attribution 4.0 International license (CC-BY 4.0) Data are available under the terms of the Creative Commons Attribution 4.0 International license (CC-BY 4.0)

## Footnotes

^1^
This differs from Gregory and Jackson (2017) who displayed colour patches for 100ms. However, when piloting our study, participants perceived this duration to be too short such that we extended the duration to 150ms.

## Software availability statement

Software used in the experiment is available from:

• Source code available from: https://github.com/elegg/gaze-cueing-wm-verbal-experiment
• Archived software: https://doi.org/10.5281/zenodo.18294236
• License: MIT License

Software used for analysis and visualisations is available from:

• Source code available from: https://github.com/elegg/gaze-cueing-memory-verbal-analysis
• Archived software available from: https://doi.org/10.5281/zenodo.18294571
• License: MIT License

### Extended data

Zenodo: Extended Data From “Gaze improves working memory for verbal items”, https://doi.org/10.5281/zenodo.18294406 ( Legg *et al.*, 2026b).

This project contains the following data:

• Extended Data - Gaze-WM.docx (a word document containing: 1. an example consent form questionnaire, 2. an example of the instructions – text and image - presented to participants in Experiment 1, 3. an example of the text presented to the participant in the debrief section of the experiment, 4. an example of the instructions presented to participants in the arrow group of Experiment 2 and, 5. an example of the instructions presented to participants in the gaze group of Experiment 2).

Data are available under the terms of the Creative Commons Attribution 4.0 International license (CC-BY 4.0)
