# Supplementary material for: Gaze cues and arrow cues modulate accuracy of recall of verbal items in a working memory task
Source: Open Res Eur. 2026 Feb 26;5:355. Originally published 2025 Nov 20. [Version 2] doi: 10.12688/openreseurope.21720.2 (PMC13188215; doi:10.12688/openreseurope.21720.2)
Supplement: Peer review history

# Reviewer report: Reviewer response for version 2

**DOI:** 10.21956/openreseurope.24771.r70371

**Published:** 2026-03-14

**Authors:** Samantha Gregory

**Affiliations:** 1 University of Salford, Salford, England, UK

The authors have addressed my comments, and I have found the results of study 2 to be enlightening. The idea around the multiple arrows being the reason for the result in study 2 is an interesting one. This definitely adds salience, as does the transition from box to arrow, so I agree with this salience interpretation. I also appreciated the replication of study 1s gaze result in study 2. 

 I recommend that the manuscript be accepted. 

If applicable, is the statistical analysis and its interpretation appropriate?

Yes

Are all the source data and materials underlying the results available?

Yes

Is the study design appropriate and is the work technically sound?

Yes

Is the work clearly and accurately presented and does it engage with the current literature?

Partly

Are the conclusions drawn adequately supported by the results?

Partly

Are sufficient details of methods and analysis provided to allow replication by others?

Yes

Reviewer Expertise:

Social gaze cuing, attention, working memory

I confirm that I have read this submission and believe that I have an appropriate level of expertise to confirm that it is of an acceptable scientific standard.

# Reviewer report: Reviewer response for version 2

**DOI:** 10.21956/openreseurope.24771.r70369

**Published:** 2026-03-05

**Authors:** Jari Hietanen

**Affiliations:** 1 Tampere University, Tampere, Finland

I congratulate the authors for carrying out a new experiment. As we see, the results were rather revealing. I have only two comments. 

 The first is very minor. In Introduction, fourth paragraph, second sentence: "For recall of items stored in long-term memory, apart from Dodd *et al.* (2012), only one study investigated the effects of cueing". >>  "..the effects of gaze cuing".

 In General Discussion, the authors discuss about the possible reasons for conflicting results regarding the effects of arrow cuing (in paragraph starting " Gregory *et al.* (2022) proposed the increased ...". The authors suggest that, perhaps, one reason for their finding a cueing effect also for arrows was related to the number of different version of their arrow cues. This might be so. But, in my mind, another possibility is related to the visual appearance of their neutral and directional symbolic cues. The change from a square (neutral) to an arrow (directional) is visually very salient. Perhaps more salient than in the previous studies not reporting an effect for arrow cues. So this could be a reason for the present findings also, and the authors might want to bring up this possibility too in their Discussion. This is just a suggestion.

 In sum, I am happy with the present version of the manuscript and have no further comments.

If applicable, is the statistical analysis and its interpretation appropriate?

Yes

Are all the source data and materials underlying the results available?

Yes

Is the study design appropriate and is the work technically sound?

No

Is the work clearly and accurately presented and does it engage with the current literature?

Partly

Are the conclusions drawn adequately supported by the results?

No

Are sufficient details of methods and analysis provided to allow replication by others?

Partly

Reviewer Expertise:

Cognitive psychology/attention orienting by gaze cuing

I confirm that I have read this submission and believe that I have an appropriate level of expertise to confirm that it is of an acceptable scientific standard.

# Reviewer report: Reviewer response for version 2

**DOI:** 10.21956/openreseurope.24771.r70368

**Published:** 2026-03-04

**Authors:** Krzysztof Cipora

**Affiliations:** 1 Copernicus Center for Interdisciplinary Studies, Jagiellonian University in Krakow, Krakow, Poland

I think the authors have done a decent job revising the manuscript and most of my concerns are properly addressed. I think adding the second experiment was a great choice. Although the second experiment changes the conclusions quite dramatically, it is very informative.

 It has now become clear why the idea of facial cuing arose in the first place, when specificities of the effect above and beyond effects of arrow cues are explained. This might be helpful to several readers.

 However, there are still a couple of points I would like the authors to address.

 1. Power analysis for Experiment 1. Where did the assumed effect size of d = 0.50 come from? In psychology it is rather a large effect, hence a word of justification would be useful. For instance, Brysbaert (refer to reference 1) as the best guess for an effect size in psychology proposes d = 0.40 and provides extensive justification for that. It is fine to be interested in larger effects only, but some explanation would help the reader understand this choice.

 2. Figures 2 and 4. I know it may be a matter of taste, but you might consider presenting the datapoints along with the boxplots. It is a matter of displaying 34 dots per condition so the figure should not be too overcrowded and shows the exact distribution of the data.

 3. Effect of faces, effect of arrows and whether they affect spatial attention or working memory. In Discussion of Experiment 1 you write: “However, in light of the debate about whether such an improvement of recall may be based on spatial attention or is directly related to changes in working memory itself and in light of previous studies highlighting that the effect for non-verbal items may be specific to social, gaze cues, (Dodd et al., 2012; Gregory & Jackson, 2017, 2019; Ishikawa & Yoshioka, 2025) it was necessary to test to what extent the effect observed in Experiment 1 was specific to the gaze cue.” I am not sure whether I can follow the argument here. To my reading you are claiming that presence of the effect for faces but not for arrows implies that working memory is engaged, while presence of both effects implies attentional effects. I am asking myself why such dissociation of effects of facial cues and arrows would indicate the role of working memory. Invalid facial cues might well be preventing the encoding of the information, so the working memory might not have obtained the sufficiently accurate material in the first place. For this argument to hold you would need to demonstrate that the material was perceived similarly in both conditions and the difference originates from the effects on working memory specifically. If I do not get the point, you might consider that some of your future readers do not get it either, and consider explaining it in a little bit more detail.

 You acknowledge a possibility that the effect takes place in attention rather than working memory in the first paragraph of the general discussion. Nevertheless, I suggest reading the paper carefully to check for consistency in this. As for now, for instance potentially problematic claims can be found in the conclusion of the abstract.

 4. Power analysis for Experiment 2. Which effect do you specifically mean? The interaction showing no effect for arrows and an effect of faces? Be more specific.

If applicable, is the statistical analysis and its interpretation appropriate?

Partly

Are all the source data and materials underlying the results available?

Yes

Is the study design appropriate and is the work technically sound?

No

Is the work clearly and accurately presented and does it engage with the current literature?

Partly

Are the conclusions drawn adequately supported by the results?

Partly

Are sufficient details of methods and analysis provided to allow replication by others?

Yes

Reviewer Expertise:

Cognitive psychology, numerical cognition with some expertise in attention and working memory.

I confirm that I have read this submission and believe that I have an appropriate level of expertise to confirm that it is of an acceptable scientific standard, however I have significant reservations, as outlined above.

## References

[1] : How Many Participants Do We Have to Include in Properly Powered Experiments? A Tutorial of Power Analysis with Reference Tables. *Journal of Cognition* .2019;2(1) : 10.5334/joc.72 10.5334/joc.72 31517234 PMC6640316

# Reviewer report: Reviewer response for version 1

**DOI:** 10.21956/openreseurope.23498.r64662

**Published:** 2026-01-08

**Authors:** Jeanette A. Chacón-Candia

**Affiliations:** 1 University of Granada, Granada, Spain

This study aimed to investigate whether socially relevant cues modulate working memory for verbalizable information, in a similar way to what has been previously reported for colour stimuli (Gregory & Jackson, 2017). In an online experiment, participants were first presented with a central schematic face looking straight ahead, which subsequently shifted its gaze to the left or right along the horizontal axis. After 500 ms, a grid containing four letters appeared either to the left or right of the central cue. Following a retention interval indicated by a fixation cross, a single probe letter was presented. Participants were required to indicate whether the probe letter had been part of the previously presented letter grid.

 Using this paradigm, the authors examined whether working memory was enhanced when the letter grid appeared at the location cued by gaze, or whether gaze-target congruency did not influence participants’ ability to remember the letters.

 The authors found that participants were more accurate in remembering the letters when the grid appeared at the gazed-at location than when it appeared on the opposite side. Based on these results, they conclude that joint attention can enhance working memory for verbalizable stimuli, such as letters.

 Before providing my comments on the manuscript, I would like to note that I consider the investigation of social attention and its connection with other cognitive functions, such as working memory, to be an interesting and valuable area of research. It is encouraging to see how other authors are engaging in this line of work and contributing to the expansion of the literature on these interesting topic.

**General Comments and Suggestions**

 - A relevant point that I believe could strengthen the manuscript concerns the relationship between the present findings and previous work by Gregory and Jackson (2017). The authors repeatedly state that their paradigm is highly comparable to that study and explicitly build on its theoretical framework. However, an important methodological difference should be acknowledged.

 In Gregory and Jackson (2017), a direct comparison was made between socially relevant cues (eye-gaze) and non-social cues (arrows and low-level motion cues). Their central conclusion (that eye-gaze selectively modulates working memory) was explicitly based on this contrast, leading the authors to argue that the effect was driven by the social relevance of gaze. In the present study, by contrast, only socially relevant cues (eye-gaze) are included. As a result, the paradigm does not allow for a direct test of whether the observed effects are specific to social cues or could also be elicited by non-social directional cues.

 Despite this limitation, the authors draw conclusions that closely mirror those of Gregory and Jackson (2017), attributing the observed working memory benefits for verbalizable stimuli to social mechanisms such as joint attention. Without including a non-social control condition, however, these claims appear less strongly supported. Explicitly discussing this methodological difference, and tempering the theoretical conclusions, accordingly, would improve the clarity and strength of the manuscript.

 Building on this point, it may be relevant for the authors to consider recent meta-analytic evidence questioning the social specificity of the classic gaze-cueing paradigm. Specifically, Chacón-Candia et al. (2023) showed that, at a quantitative level, the classic gaze-cueing task does not produce effects that reliably differ from those elicited by non-social cues such as arrows. This evidence suggests that the attentional orienting effects observed in standard gaze-cueing paradigms may reflect domain-general mechanisms rather than uniquely social processes.

 In light of this, taking into account this literature, and maybe extending the current design to include an appropriate non-social control cue (e.g., arrows), or at least discussing this limitation explicitly, would substantially strengthen the manuscript. In particular, a qualitative comparison between social and non-social cues within the working memory task would allow the authors to more convincingly support claims regarding the role of joint attention or social relevance in modulating working memory performance.

 - Following the previous point, it may be worth expanding the theoretical background to include additional recent studies examining the relationship between social attentional orienting and working memory. Although these studies do not focus exclusively on verbal working memory or employ identical experimental paradigms, they are nevertheless highly relevant to the aims of the present work, as they address how socially relevant cues influence attention and working memory processes.

 For example, studies such as Foglino & Wykowska (2025), Nie et al. (2018), and Zhang et al. (2023) provide important evidence showing how social attention can modulate working memory performance under different task conditions. I consider that including this literature would help better situate the present findings within the broader field and strengthen the theoretical framework of the study.

**Minor comments / clarifications**

 - Out of curiosity, it would be helpful to report the age range of the participants. Given that the mean age is 38 years, this seems relatively high, maybe providing the full range would help to better characterize the sample.

 - The manuscript mentions that two participants were excluded based on the criterion of 2.5 mean absolute deviations. However, it is not entirely clear how many participants were included in the final sample after exclusions. We suggest clarifying this information explicitly in the Participants section.

 - It is not reported in the manuscript whether the hypotheses were pre-registered prior to data collection. If the study was not pre-registered, we would simply encourage the authors to consider this practice in future work, as it can enhance transparency and reproducibility.

If applicable, is the statistical analysis and its interpretation appropriate?

Partly

Are all the source data and materials underlying the results available?

Yes

Is the study design appropriate and is the work technically sound?

Partly

Is the work clearly and accurately presented and does it engage with the current literature?

Yes

Are the conclusions drawn adequately supported by the results?

No

Are sufficient details of methods and analysis provided to allow replication by others?

Yes

Reviewer Expertise:

Social Attention, Social Cognition

I confirm that I have read this submission and believe that I have an appropriate level of expertise to state that I do not consider it to be of an acceptable scientific standard, for reasons outlined above.

## References

[1] : Are there quantitative differences between eye-gaze and arrow cues? A meta-analytic answer to the debate and a call for qualitative differences. *Neuroscience & Biobehavioral Reviews* .2023;144: 10.1016/j.neubiorev.2022.104993 10.1016/j.neubiorev.2022.104993 36496190

[2] : Joint attention supports working memory when gaze cues are reliable and task-relevant. *Attention, Perception, & Psychophysics* .2026;88(1) : 10.3758/s13414-025-03163-x 10.3758/s13414-025-03163-x PMC1267261241331189

[3] : Social attention directs working memory maintenance. *Cognition* .2018;171: 10.1016/j.cognition.2017.10.025 85-94 10.1016/j.cognition.2017.10.025 29121587

[4] : When “looking at nothing” imparts something: Retrospective gaze cues flexibly direct prioritization in visual working memory. *Journal of Experimental Psychology: Human Perception and Performance* .2023;49(11) : 10.1037/xhp0001160 1407-1419 10.1037/xhp0001160 37870820

# Response

**Published:** 2026-01-19

**Authors:** Edward Legg

Dear Dr. Chacón Candia,    

 Many thanks for your thorough and helpful comments on the original version of our manuscript. We have changed the manuscript according to your comments, all of which also align with the comments by the other reviewers. This refers to two main aspects, namely the inclusion of a second experiment that tests the effects of both social cues (gaze cue) and non-social cues (arrow cue) on recall of verbal items in working memory, and the expansion of the theoretical discussions in this research field.    

 Below we provide point-by-point responses to your comments on the original version of our manuscript – for this we have pasted your comments in italics and provide our replies in plain font.  

*This study aimed to investigate whether socially relevant cues modulate working memory for verbalizable information, in a similar way to what has been previously reported for colour stimuli (Gregory & Jackson, 2017). In an online experiment, participants were first presented with a central schematic face looking straight ahead, which subsequently shifted its gaze to the left or right along the horizontal axis. After 500 ms, a grid containing four letters appeared either to the left or right of the central cue. Following a retention interval indicated by a fixation cross, a single probe letter was presented. Participants were required to indicate whether the probe letter had been part of the previously presented letter grid.* *Using this paradigm, the authors examined whether working memory was enhanced when the letter grid appeared at the location cued by gaze, or whether gaze-target congruency did not influence participants’ ability to remember the letters.* *The authors found that participants were more accurate in remembering the letters when the grid appeared at the gazed-at location than when it appeared on the opposite side. Based on these results, they conclude that joint attention can enhance working memory for verbalizable stimuli, such as letters.* *Before providing my comments on the manuscript, I would like to note that I consider the investigation of social attention and its connection with other cognitive functions, such as working memory, to be an interesting and valuable area of research. It is encouraging to see how other authors are engaging in this line of work and contributing to the expansion of the literature on these interesting topic.* *General Comments and Suggestions* *- A relevant point that I believe could strengthen the manuscript concerns the relationship between the present findings and previous work by Gregory and Jackson (2017). The authors repeatedly state that their paradigm is highly comparable to that study and explicitly build on its theoretical framework. However, an important methodological difference should be acknowledged.* *In Gregory and Jackson (2017), a direct comparison was made between socially relevant cues (eye-gaze) and non-social cues (arrows and low-level motion cues). Their central conclusion (that eye-gaze selectively modulates working memory) was explicitly based on this contrast, leading the authors to argue that the effect was driven by the social relevance of gaze. In the present study, by contrast, only socially relevant cues (eye-gaze) are included. As a result, the paradigm does not allow for a direct test of whether the observed effects are specific to social cues or could also be elicited by non-social directional cues.* *Despite this limitation, the authors draw conclusions that closely mirror those of Gregory and Jackson (2017), attributing the observed working memory benefits for verbalizable stimuli to social mechanisms such as joint attention. Without including a non-social control condition, however, these claims appear less strongly supported. Explicitly discussing this methodological difference, and tempering the theoretical conclusions, accordingly, would improve the clarity and strength of the manuscript.*  

**Response:** Thank you for these comments. We have opted to conduct a second experiment, in which we directly compared gaze cues and arrow cues. The results of this second experiment showed cueing effects on recall by both gaze cues (thus replicating our original findings) and arrow cues. We discuss these findings in the revised Discussion section, which we have now extended to also discuss methodological differences to the Gregory and Jackson (2017) but also other previous studies, and what conclusions can be drawn based on these comparisons across studies.    

*Building on this point, it may be relevant for the authors to consider recent meta-analytic evidence questioning the social specificity of the classic gaze-cueing paradigm. Specifically, Chacón-Candia et al. (2023) showed that, at a quantitative level, the classic gaze-cueing task does not produce effects that reliably differ from those elicited by non-social cues such as arrows. This evidence suggests that the attentional orienting effects observed in standard gaze-cueing paradigms may reflect domain-general mechanisms rather than uniquely social processes.*  

**Response: **Thank you for making us aware of this meta-analysis. It is particularly relevant given that our Experiment 2 finds a similar effect in both the gaze and arrow group. We have now included this meta-analysis when we relate the findings from standard detection tasks to those investigating cueing on recall in memory tasks.   

*In light of this, taking into account this literature, and maybe extending the current design to include an appropriate non-social control cue (e.g., arrows), or at least discussing this limitation explicitly, would substantially strengthen the manuscript. In particular, a qualitative comparison between social and non-social cues within the working memory task would allow the authors to more convincingly support claims regarding the role of joint attention or social relevance in modulating working memory performance.*    

**Response: **Thank you for the suggestions. We felt it would be most appropriate to run a second experiment with a non-social control. The results of this second experiment showed cueing effects on recall by both gaze cues (thus replicating our original findings) and arrow cues, which are discussed further in the revised Discussion section, which has now also been expanded.    

*- Following the previous point, it may be worth expanding the theoretical background to include additional recent studies examining the relationship between social attentional orienting and working memory. Although these studies do not focus exclusively on verbal working memory or employ identical experimental paradigms, they are nevertheless highly relevant to the aims of the present work, as they address how socially relevant cues influence attention and working memory processes.* *For example, studies such as Foglino & Wykowska (2025), Nie et al. (2018), and Zhang et al. (2023) provide important evidence showing how social attention can modulate working memory performance under different task conditions. I consider that including this literature would help better situate the present findings within the broader field and strengthen the theoretical framework of the study.*    

**Response:**Thank you for highlighting these studies. Originally, we were constrained by a word limit on brief reports, however we have now expanded both our Introduction and Discussion, and we have included these three studies too.    

*Minor comments / clarifications*    

*- Out of curiosity, it would be helpful to report the age range of the participants. Given that the mean age is 38 years, this seems relatively high, maybe providing the full range would help to better characterize the sample.*    

**Response: **We have now provided information about the age range along with the mean and standard deviation that we reported previously.    

*- The manuscript mentions that two participants were excluded based on the criterion of 2.5 mean absolute deviations. However, it is not entirely clear how many participants were included in the final sample after exclusions. We suggest clarifying this information explicitly in the Participants section.*    

**Response: **We have now added information about final sample sizes used in the analysis for both our experiments.    

*- It is not reported in the manuscript whether the hypotheses were pre-registered prior to data collection. If the study was not pre-registered, we would simply encourage the authors to consider this practice in future work, as it can enhance transparency and reproducibility.*    

**Response:**Thank you for the suggestion, and we agree on the usefulness of pre-registrations for transparency of research. We had not pre-registered the original study, which was the outcome of a student project (not that students projects cannot or should not be pre-registered, indeed we teach students about preregistrations), which is why we also did not think of pre-registering the second experiment simply because we did not think about it at the time. At the time we ran the second experiment we had not yet received your review (it was clear from the previous reviews that running an experiment with a non-social control was the best course of action). We will take this as a good reminder to incorporate pre-registrations into our workflow.

# Reviewer report: Reviewer response for version 1

**DOI:** 10.21956/openreseurope.23498.r64660

**Published:** 2025-12-29

**Authors:** Krzysztof Cipora

**Affiliations:** 1 Copernicus Center for Interdisciplinary Studies, Jagiellonian University in Krakow, Krakow, Poland

 In the paper the authors show how cuing with a directed gaze affects working memory measured with accuracy of probe recognition depending on whether the stimuli set was presented on the cued or non-cued side. The authors report better accuracy for cued than non-cued stimuli, while there is no difference in terms of reaction times.

 The paper reads very well and clearly communicates what the authors have done (also followed by shared materials), what results they obtained and how they interpret the results. Thus I can see a merit in its publication at some point. At the same time, I would like to raise rather critical points when it comes to interpretation of the results both in terms of joint attention and in terms of crucial role of working memory.

 Joint attention aspect: In studies demonstrating cuing paradigm (more or less along the lines of M. Posner model of attention), the basic stimulus used to guide participants’ attention (in a so-called endogenous paradigm) have been arrows. They facilitate detection of a stimulus presented laterally on the cued side etc. See e.g., a rather classic paper by Raz & Buhle 2006 ( https://doi.org/10.1038/nrn1903). See also a recent review on the cuing with eye gaze: https://doi.org/10.1037/bul0000353

 To be able to show that the effect is due to social cues in particular (and joint attention) one would need to either (a) dissociate this effect from effect caused by non-social stimuli (e.g., arrows), (b) provide a compelling theoretical argument that the effect of cuing in related to joint attention event without the social aspect of the cue. The latter to my understanding is not so obvious - even without social aspect there is an adaptive advantage of attention functioning this way, and Posner’s model provides quite a lot of such arguments.

 Working memory aspect: if we assume that cuing mechanism takes place, it implies that spatial attention is directed towards a specific location and tuned to perceive the stimuli appearing in that location. In case of incongruent trial, the attention is directed elsewhere, it needs to be re-allocated to the place where the stimulus is actually presented. Only following that process the information about the stimulus is gathered. Given the stimulus presentation time is rather limited (150ms), the re-orienting of attention might be taking some extra time and resources, leaving less time to properly encode the stimulus. In short: the effects do not necessarily originate from the functioning of working memory.

If applicable, is the statistical analysis and its interpretation appropriate?

Partly

Are all the source data and materials underlying the results available?

Yes

Is the study design appropriate and is the work technically sound?

No

Is the work clearly and accurately presented and does it engage with the current literature?

Partly

Are the conclusions drawn adequately supported by the results?

Partly

Are sufficient details of methods and analysis provided to allow replication by others?

Yes

Reviewer Expertise:

Cognitive psychology, numerical cognition with some expertise in attention and working memory.

I confirm that I have read this submission and believe that I have an appropriate level of expertise to state that I do not consider it to be of an acceptable scientific standard, for reasons outlined above.

## References

[1] : Typologies of attentional networks. *Nature Reviews Neuroscience* .2006;7(5) : 10.1038/nrn1903 367-379 10.1038/nrn1903 16760917

[2] : Visual attentional orienting by eye gaze: A meta-analytic review of the gaze-cueing effect. *Psychological Bulletin* .2021;147(12) : 10.1037/bul0000353 1269-1289 10.1037/bul0000353 35404635

# Response

**Published:** 2026-01-19

**Authors:** Edward Legg

Dear Prof. Cipora,    

 Thank you for the thoughtful and detailed comments to the original version to our manuscript.    In line with your and the other reviewers’ comments, we opted to conduct a second experiment in which we tested two new groups of participants: one with gaze cues as the orienting cue, and one with arrow cues as the orienting cue. The results show that recall of verbal items was modulated by gaze cues (thus replicating the findings of our original experiment – now experiment 1) but also by arrow cues.    

 In line with your suggestions to expand the discussion of conceptual questions and possible mechanisms, we have now expanded both the introduction and discussion sections. In the latter, we interpret the observed findings in line with orienting cues having shifted participants’ spatial attention.    

 Below we provide point-by-point responses to your comments on the original version of our manuscript – for this we have pasted your comments in italics and provide our replies in plain font.    

*In the paper the authors show how cuing with a directed gaze affects working memory measured with accuracy of probe recognition depending on whether the stimuli set was presented on the cued or non-cued side. The authors report better accuracy for cued than non-cued stimuli, while there is no difference in terms of reaction times.* *The paper reads very well and clearly communicates what the authors have done (also followed by shared materials), what results they obtained and how they interpret the results. Thus I can see a merit in its publication at some point. At the same time, I would like to raise rather critical points when it comes to interpretation of the results both in terms of joint attention and in terms of crucial role of working memory.* *Joint attention aspect: In studies demonstrating cuing paradigm (more or less along the lines of M. Posner model of attention), the basic stimulus used to guide participants’ attention (in a so-called endogenous paradigm) have been arrows. They facilitate detection of a stimulus presented laterally on the cued side etc. See e.g., a rather classic paper by Raz & Buhle 2006 (https://doi.org/10.1038/nrn1903). See also a recent review on the cuing with eye gaze: https://doi.org/10.1037/bul0000353* *To be able to show that the effect is due to social cues in particular (and joint attention) one would need to either (a) dissociate this effect from effect caused by non-social stimuli (e.g., arrows), (b) provide a compelling theoretical argument that the effect of cuing in related to joint attention event without the social aspect of the cue. The latter to my understanding is not so obvious - even without social aspect there is an adaptive advantage of attention functioning this way, and Posner’s model provides quite a lot of such arguments.*    

**Response:** Thank you for these comments. We agree that a non-social control is a critical step toward showing that a cueing effect is specific to social stimuli. We have now conducted a second experiment which we report in the revised version of the manuscript. The results of this second experiment replicated the effect of gaze cues on recall for verbal items but also showed an equivalent effect of arrow cues on recall.    

*Working memory aspect: if we assume that cuing mechanism takes place, it implies that spatial attention is directed towards a specific location and tuned to perceive the stimuli appearing in that location. In case of incongruent trial, the attention is directed elsewhere, it needs to be re-allocated to the place where the stimulus is actually presented. Only following that process the information about the stimulus is gathered. Given the stimulus presentation time is rather limited (150ms), the re-orienting of attention might be taking some extra time and resources, leaving less time to properly encode the stimulus. In short: the effects do not necessarily originate from the functioning of working memory.*    

**Response: **Thank you for raising this point. We agree and this is particularly pertinent in light of our second experiment because in the absence of a difference between the gaze and arrow groups, it is possible that the cueing effects are explained by a shift in spatial attention at encoding. We discuss this in detail in the Discussion section of the revised manuscript.

# Reviewer report: Reviewer response for version 1

**DOI:** 10.21956/openreseurope.23498.r64661

**Published:** 2025-12-27

**Authors:** Samantha Gregory

**Affiliations:** 1 University of Salford, Salford, England, UK

The manuscript presents a single study investigating whether gaze cues affect working memory for verbalizable items. The findings show an effect of gaze on memory for these items.

 The study is justified and the findings as they are presented are clear and well presented. However, I have a few concerns.

 While the basis of the study being Dodd et al 2012, and Gregory and Jackson 2017 was clear,  there was a limited literature base drawn upon, and while this is a brief report some consideration of more recent findings in the area would be appropriate.

 It is also notable that there was no control comparison conducted, in previous gaze cuing work mentioned, such as Gregory and Jackson, Dodd et al, and Bayliss et al 2006, an arrow cue was used as a non-social control stimuli. This comparison is required to be able to claim a social cuing effect. I believe this gap should at least be considered in the manuscript, even if not directly tested.

If applicable, is the statistical analysis and its interpretation appropriate?

Yes

Are all the source data and materials underlying the results available?

Yes

Is the study design appropriate and is the work technically sound?

Yes

Is the work clearly and accurately presented and does it engage with the current literature?

Partly

Are the conclusions drawn adequately supported by the results?

Partly

Are sufficient details of methods and analysis provided to allow replication by others?

Yes

Reviewer Expertise:

Social gaze cuing, attention, working memory

I confirm that I have read this submission and believe that I have an appropriate level of expertise to confirm that it is of an acceptable scientific standard, however I have significant reservations, as outlined above.

# Response

**Published:** 2026-01-19

**Authors:** Edward Legg

Dear Dr. Gregory,  

 Thank you for your comments to the original version to our manuscript, which helped us in the revision process.   Based on your comments – all of which also align with the other reviewers’ comments – we have changed two main things in the revised manuscript: first, we have expanded the overview of the research field and the discussions within both in the Introduction and Discussion sections, and second, we have conducted and now report a second experiment, in which we tested the effect on recall of both gaze cues and arrow cues.   This experiment revealed that both gaze and arrow cues improved participants’ performance in the working memory task, such that in the discussion we now include a possible and most parsimonious interpretation based on shifts in spatial attention.   Below we provide point-by-point responses to your comments on the original version of our manuscript – for this we have pasted your comments in italics and provide our replies in plain font.

*The manuscript presents a single study investigating whether gaze cues affect working memory for verbalizable items. The findings show an effect of gaze on memory for these items.* *The study is justified and the findings as they are presented are clear and well presented. However, I have a few concerns.* *While the basis of the study being Dodd et al 2012, and Gregory and Jackson 2017 was clear, there was a limited literature base drawn upon, and while this is a brief report some consideration of more recent findings in the area would be appropriate.*  

 We thank you for the suggestion to expand the Introduction. We were initially constrained by the word limit of the brief report, but we have now expanded the Introduction section of the revised manuscript, such that a more thorough overview of the literature and the debates within it are provided to the reader.  

*It is also notable that there was no control comparison conducted, in previous gaze cuing work mentioned, such as Gregory and Jackson, Dodd et al, and Bayliss et al 2006, an arrow cue was used as a non-social control stimuli. This comparison is required to be able to claim a social cuing effect. I believe this gap should at least be considered in the manuscript, even if not directly tested.*  

 We have now conducted a second experiment which we report in the revised version of the manuscript, in which we replicated the effect of gaze cues on recall for verbal items but also found an equivalent effect of arrow cues on recall. In relating these results to previous literature, we have now also expanded the Discussion section.

# Reviewer report: Reviewer response for version 1

**DOI:** 10.21956/openreseurope.23498.r64659

**Published:** 2025-11-25

**Authors:** Jari Hietanen

**Affiliations:** 1 Tampere University, Tampere, Finland

The manuscript describes a single study investigating the effect of gaze cuing on working memory, specifically for retention of verbal information (a matrix of 4 letters). The results showed that the participants were more accurate in judging whether a target letter (presented 1000 ms after the presentation of the matrix) had been among letters in the matrix when the matrix appeared at the gaze-at location than when it appeared at the opposite location. The authors interpreted the results to show that “joint attention can improve working memory for verbalizable content such as letters”.

 As a basis for their study, the authors refer to two previous studies which investigated the effects of gaze cuing on long-term retention of verbal stimuli (Dodd et al., 2012) and the effects of gaze cuing on short-term (working memory) retention of non-verbal visual information (colors) (Gregory & Jackson, 2017).  The authors motivate their study by arguing that they aim to investigate whether gaze cuing (or joint attention as they call it) improves also verbal working memory performance.

 The aim of the study is justified. However, if the authors aim to show that joint attention improves verbal working memory, they should be able to show that the reported findings are not just reflecting general attentional effects. In studies by both Dodd et al. (2012) and by Gregory & Jackson (2017) this was achieved by showing that attention cuing by symbolic cues (arrows) did not result in better memory performance for cued vs. uncued simuli. I am somewhat surprised that the authors of the present manuscript do not describe this aspect of those two previous studies at all, nor they discuss the potential issue related to attentional vs. memory effects behind their findings in their discussion either. By the way, there is also a more recent study by Ishikawa and Yoshioka (2025) in which they also used the same approach (i.e. comparing gaze cuing vs arrow cuing).

 Thus, I am afraid but the present study cannot provide convincing evidence that joint attention improves verbal working memory performance.

 Otherwise, the experiment appeared to be carefully conducted and the results were appropriately analyzed.

 As a minor issue, in Methods, the authors could mention that, on each trial, the letters in the matrix were randomly selected among the used consonants. I take that this was the case. Also, I suppose that the target letter was one of those presented in the matrix on half of the trials, and that this manipulation was orthogonal to that of the cuing (i.e. cued vs uncued).

 Reference

 Ishikawa, M., Yoshioka, A. Gaze cues facilitate incidental learning in children aged 7–10 years, but arrow cues do not. *Psychon Bull Rev* **32**, 1712–1721 (2025). https://doi.org/10.3758/s13423-025-02657-x

If applicable, is the statistical analysis and its interpretation appropriate?

Yes

Are all the source data and materials underlying the results available?

Yes

Is the study design appropriate and is the work technically sound?

No

Is the work clearly and accurately presented and does it engage with the current literature?

Partly

Are the conclusions drawn adequately supported by the results?

No

Are sufficient details of methods and analysis provided to allow replication by others?

Partly

Reviewer Expertise:

Cognitive psychology/attention orienting by gaze cuing

I confirm that I have read this submission and believe that I have an appropriate level of expertise to state that I do not consider it to be of an acceptable scientific standard, for reasons outlined above.

# Response

**Published:** 2026-01-19

**Authors:** Edward Legg

Dear Prof. Hietanen, Thank you for the thoughtful comments to the original version to our manuscript. In light of your and the other reviewers’ comments, we have opted to conduct a second experiment. Our intention had been to provide data and a short report regarding the first step in testing whether orienting cues affect recall in a working memory task for verbal content, without necessarily going further into what may underlie this effect. However, in light of your and the other reviewers’ comments, all of which we agree with, we have opted to conduct a second experiment. In this second experiment, we tested two new groups of participants, one for whom the orienting cue was a gaze cue (direct replication of Experiment 1., i.e., the study reported in the original version of our manuscript) and one for whom the orienting cue was an arrow cue. Here, we found that both gaze and arrow cues improved participants’ performance in the working memory task. Consequently, we have expanded our discussion to include an interpretation based on shifts in spatial attention (which provides the most likely and parsimonious explanation for our pattern of results), but where we also briefly discuss avenues for future research that are opened up by these results. Below we provide point-by-point responses to your comments on the original version of our manuscript – for this we have pasted your comments in italics and provide our replies in plain font.

*The manuscript describes a single study investigating the effect of gaze cuing on working memory, specifically for retention of verbal information (a matrix of 4 letters). The results showed that the participants were more accurate in judging whether a target letter (presented 1000 ms after the presentation of the matrix) had been among letters in the matrix when the matrix appeared at the gaze-at location than when it appeared at the opposite location. The authors interpreted the results to show that “joint attention can improve working memory for verbalizable content such as letters”.* *As a basis for their study, the authors refer to two previous studies which investigated the effects of gaze cuing on long-term retention of verbal stimuli (Dodd et al., 2012) and the effects of gaze cuing on short-term (working memory) retention of non-verbal visual information (colors) (Gregory & Jackson, 2017). The authors motivate their study by arguing that they aim to investigate whether gaze cuing (or joint attention as they call it) improves also verbal working memory performance.* *The aim of the study is justified. However, if the authors aim to show that joint attention improves verbal working memory, they should be able to show that the reported findings are not just reflecting general attentional effects. In studies by both Dodd et al. (2012) and by Gregory & Jackson (2017) this was achieved by showing that attention cuing by symbolic cues (arrows) did not result in better memory performance for cued vs. uncued simuli. I am somewhat surprised that the authors of the present manuscript do not describe this aspect of those two previous studies at all, nor they discuss the potential issue related to attentional vs. memory effects behind their findings in their discussion either. By the way, there is also a more recent study by Ishikawa and Yoshioka (2025) in which they also used the same approach (i.e. comparing gaze cuing vs arrow cuing).*

 Thank you for the comment – we have now conducted a second experiment which we report in the revised version of the manuscript, and which is described in more detail also in our general reply to the reviewers’ comments. The results of this second experiment – whereby we replicate the effect of gaze cues on recall for verbal items but also found an equivalent effect of arrow cues on recall – are now also discussed in terms of the most likely explanation through general attentional effects. We are also grateful for the information about the recent Ishikawa and Yoshioko (2025) study, which we had missed, and have now included it in the Introduction of the revised manuscript.

*Thus, I am afraid but the present study cannot provide convincing evidence that joint attention improves verbal working memory performance.* *Otherwise, the experiment appeared to be carefully conducted and the results were appropriately analyzed.* *As a minor issue, in Methods, the authors could mention that, on each trial, the letters in the matrix were randomly selected among the used consonants. I take that this was the case. Also, I suppose that the target letter was one of those presented in the matrix on half of the trials, and that this manipulation was orthogonal to that of the cuing (i.e. cued vs uncued).*  

 Thank you for alerting us that we missed to provide this information in the Methods of the original manuscript - we have now added information about the randomisation. The reviewer is correct that the target letter had been present in the array of consonants on half of trials and we have now described this in the Methods section of the revised manuscript.
